# Game Meat Consumption—Conscious Choice or Just a Game?

**DOI:** 10.3390/foods9101357

**Published:** 2020-09-24

**Authors:** Katarzyna Niewiadomska, Małgorzata Kosicka-Gębska, Jerzy Gębski, Krystyna Gutkowska, Marzena Jeżewska-Zychowicz, Marianna Sułek

**Affiliations:** Institute of Human Nutrition Sciences, Warsaw University of Life Sciences (SGGW-WULS), Nowoursynowska 159C, 02-776 Warsaw, Poland; malgorzata_kosicka_gebska@sggw.edu.pl (M.K.-G.); jerzy_gebski@sggw.edu.pl (J.G.); krystyna_gutkowska@sggw.edu.pl (K.G.); marzena_jezewska_zychowicz@sggw.edu.pl (M.J.-Z.); marianna_sulek@sggw.edu.pl (M.S.)

**Keywords:** game meat, choice motives, quality perception, quality attributes

## Abstract

Game meat is constantly present on the European meat market, but a limited number of consumers are interested in its consumption. Considering the unique features of wild animal meat, we should explore what pushes consumers to include it in their diet. To identify the motives determining the choice of game meat, a quantitative survey based on the computer-assisted telephone interview (CATI) method was conducted among 450 participants. The statistical analysis based on the logistic regression model allowed us to assess the significance of emotional motives leading to game eating and to investigate the importance of the rational motives related to the quality attributes of game for consumers. It was shown that rational motives influence the consumers’ choice more than emotional factors, while the most important motives are connected with healthcare issues. Consumers, for whom the crucial attributes of quality are taste, nutritional value, and low fat content, constitute a group that might more often include game in their diet in the future. Among the emotional motives, the familiarity, described as a feeling of knowing the product, also has a statistically significant impact on the consumers’ choice. The results obtained may be useful for academic theoreticians and market experts as well.

## 1. Introduction

Consumers’ choices are triggered by different motives perceived as the permanent predispositions directing human behavior [1,2]. Different motives can simultaneously lead to achieving one specific goal [3]. Depending on the effect they cause, two groups of motives can be defined: emotional motives (leading to a comfortable feeling or good mood caused by food consumption) and rational motives (leading to satisfaction related to the consumption of food with specific properties and expected quality) [4]. Both groups of motives, emotional and rational ones, determine the food choice and the frequency of its consumption. As rational motives were mentioned, the food quality term should be explained. 

Quality is perceived as one of the most important factors determining consumers’ behavior, even though no comprehensive definition of this concept has been developed so far [5,6,7]. The concept of quality is widely used, but the term is interpreted differently by consumers [8,9,10]. Meat consumers indicate meat freshness, taste, odor, general appearance, labeling and origin as the crucial quality features [11]. According to the generally accepted definition, food quality could be explained as the totality of features and characteristics of food satisfying customers [12]. 

Considering the number of common nutrition mistakes and their consequences, the investigation of the meaning of motives that determine consumers’ choices seems important in the perspective of future dietary behavior modification. Nutritionists recommend implementing changes to balance out the intake of carbohydrates, salt and fat, as well as fruit and vegetable consumption [13]. Moreover, meat intake should be controlled and regulated since the correlation between a high level of fat and processed meat consumption and the occurrence of diet-related diseases, such as obesity, cardiovascular disease, and cancer, has been revealed [14,15,16,17,18,19,20]. Recently, a strong disproportion between the recommended meat consumption level and the actual intake has been observed. The European and U.S. authorities advise people to eat no more than 500 g of red and processed meat weekly [21,22]. However, studies show that the red meat intake in North America, Latin America, and Europe is 300–600% higher than the recommended levels [23]. 

To prevent the occurrence of diet-related diseases, the authorities recommend a change in the composition of the daily diet by limiting meat consumption in favor of increasing the share of plant products [24,25]. A change in the plant and meat proportion in the diet seems hard to implement in societies with a strong tradition of meat consumption [26]. In particular, in Western European countries, consumers are not interested in changing their meat-eating patterns [27,28]. They refuse to try meat substitutes and are not used to a plant-based diet [29,30].

Considering the mentioned obstacles, finding an alternative solution that can help to improve the diet is needed. One of the ideas is to encourage people to try other types of meat, characterized by different nutrient compositions, for example, a smaller proportion of saturated fatty acids in the general chemical composition or the amino acid composition most beneficial for human health, versus the most often consumed meat. In countries with strongly rooted hunting traditions and environmental conditions favorable for hunting, game meat could become an alternative for meat produced via intensive livestock farming [31,32,33,34,35]. 

The concept of game is not clearly defined in the literature [36]. In Europe, the term game refers to edible parts (and blood) of animals of species recognized as wild, according to the legal regulations of the country [37]. In most European countries, game meat is mainly derived from red deer, roe deer, fallow deer, wild boar, wild rabbit, and wild birds of different species. A common trait characterizing wild animal meat is a number of features that have a positive impact on human health and body functioning, which is a result of its good nutrient composition (proteins, unsaturated fatty acids, vitamins, macro- and microelements) in comparison with livestock meat [34,36,38,39]. Though the high nutritional value of game meat has been documented, its consumption in Europe stays at a low level [40,41,42,43,44,45,46,47,48]. Game meat is perceived as a prestigious and sophisticated food and the game market is still a niche, hardly attainable for consumers [49,50,51].

In the process of reviewing the literature, it was found that the number of studies regarding the game meat choice motives of European consumers is low. What is more, no data show the impact of the particular attributes of game on the frequency of its consumption. Due to the beneficial characteristics of this type of meat, it seems reasonable to find an answer to the question: what can lead consumers to include game meat into their diet? Therefore, the purpose of this study was twofold: (1) to assess the significance of emotional motives leading to game meat choice, based on Food Choice Questionnaire items [52], and (2) to expose the rational motives related to attributes of the quality of game, which are important for Polish consumers. The relation between crucial motives and the frequency of consumption of game meat was also verified. It needs to be emphasized that the purpose of this work is not to encourage consumers to eat more meat in general, but to inspire them to try game, a lean and flavorful type of meat, as an alternative protein source in the diet. 

## 2. Materials and Methods

### 2.1. Ethical Approval

The Ethics Committee of the Faculty of Human Nutrition and Consumer Science, Warsaw University of Life Sciences (SGGW-WULS), appointed on the basis of Regulation No. 27 of the SGGW Rector of 5 May 2016, approved the protocol for the impact of selected conditions on consumers’ behavior towards game meat on 27 June 2016, Resolution No. 03/2016, as consistent with the guidelines laid down in the Declaration of Helsinki. Informed consent was provided by participants.

### 2.2. Research Approach and Sampling

The data were collected in 2016 in a nationwide survey using computer-assisted telephone interview (CATI) technology. The sample was gathered using the random dialing and validation of telephone numbers. The sample size reflected the demographic structure of the population in 16 country regions (voivodeships) in accordance with the public data of the Central Statistical Office (GUS). The bottom limit of the respondent age was determined as 25 years based on the GUS data, which suggests that at the age of 25 the average consumer sets up a separate household, and thus begins to make independent purchasing decisions. The upper age limit has not been determined. The selection of the sample was targeted to people consuming game (now or in the past). Due to the limited literature background, no additional criteria related to consumption were implemented. For analysis purposes, the participants (*N* = 450) were divided into two groups due to the frequency of their consumption of game: ‘heavy users’ (eating game at least a few times a year) and ‘light users’ (eating game less often than once every six months). The characteristics of the study sample are presented in Table 1.

### 2.3. Questionnaire Content and Pre-Testing

The tool used in the study was an original questionnaire with a high degree of standardization. It consisted of 21 questions regarding consumers’ behavior, motives and attitudes towards game. Moreover, the socio-economic characteristics of respondents were verified. The questionnaire was tested in a pilot study through personal interviews with 33 respondents in order to identify and eliminate potential problems.

In the paper, 3 aspects related to game consumption were explored: (1) emotional motives that influence the consumers’ decisions based on the Food Choice Questionnaire adjusted for the survey, (2) rational motives determining the perception of quality features of game meat and its choice, (3) the impact of emotional and rational motives on the frequency of eating meat derived from wild animals.

### 2.4. Measurement and Scaling

By modifying the Food Choice Questionnaire (FCQ) designed by Steptoe [52], we created a 32-item questionnaire, taking into account the unique character of game meat. Using the tool, we were able to define the emotional motives important for a consumer who includes game meat in their diet. Respondents were asked to rate the importance of the statements starting from “It is important to me that game meat in my diet …” (Table 2). The significance of each motive was measured using a 5-point scale ranging from “not important at all” (1) to “extremely important” (5).

The meaning of rational motives in the food choice process was tested by an analysis of the consumers’ perception of game meat quality attributes (Table 3). Respondents were asked to express their opinion about 18 attributes using the sentence: “When I think about the game meat available on the market, as the important feature determining the meat quality I consider…”. Each motive was measured using a 5-point scale ranging from “not important at all for me (1) to “extremely important for me” (5). Finally, the relation between the importance of quality features and the frequency of the consumption of game meat was examined.

### 2.5. Statistical Analyses

Completed questionnaires were coded by the research agency. The statistical packages SAS 9.4 (SAS Institute, Cary, NC, USA) and PS Imago 5.0 (Predictive Solutions Sp. z o.o., Krakow, Poland) were used for statistical analyses. As a part of primary data processing, frequency analysis and contingency tables were used. Descriptive statistics were also performed. The independent chi-squared test was used to investigate the relationship between selected variables and game meat consumption frequency; *p* < 0.05 was considered significant.

Logistic regression analysis was used to develop a prognostic model, exposing the emotional and rational motives that lead a consumer to include game meat in a varied diet more often. The dependent variable in the model was the frequency of game consumption. The dependent variable had a dichotomous scale: “heavy users”—eat game meat at least twice a year/“light users”—eat game meat less than twice a year. The explanatory variables were the participants’ opinions on the importance of the FCQ items and attributes of the quality of game. The predicted level of the dependent variable was described as a ‘heavy user’. The selection of regressors based on their importance in the model was performed using a step selection (stepwise forward). Only statistically significant independent variables at *p* < 0.05 were left in the regression model (Table 4).

The assessment of the adherence of the applied model was carried out with the use of classic tools for the logistic regression model (Table 5). The quality of the developed model was assessed by the pseudo-R^2^ (Cox and Snell) model fit, which is 0.191, and its maximum scaled value (Nagelkerke) is 0.223. A test was conducted on the coefficients of the model, which hypothesized ‘H0: all the parameters in the model are equal to 0′. This hypothesis was rejected at every level of significance, both for the likelihood ratio and the Wald statistics. The Hosmer and Lemeshow test, based on percentiles, calculated the probabilities of all the observations divided into 10 groups. Pearson’s chi-squared-statistics analyzed the differences between the observed and expected value. The high *p*-value equal to 0.995 did not allow the rejection of the H0 hypothesis, assuming the agreement of the theoretical and empirical values; therefore, it confirmed that the model was appropriately adjusted. An assessment of the adequacy of the model was carried out at a standard significance level of 0.05 [53,54]. When analyzing predicted associations of probabilities and observed responses, 69.9% of the cases were classified correctly. Statistic c for the model is 0.699, which proves that the model predicts results correctly and with a fairly high probability [55].

## 3. Results

As previous studies have shown, consumers show willingness to increase the share of game in their daily diet [32,51,56]. The presented study has shown that almost 74% of Polish game consumers eat wild animal meat less frequently than once a month, while 7% of respondents have a game meat dish on the table a few times a week.

Among the attributes describing the quality of game, ‘taste’ had the greatest impact on the frequency of its consumption (Wald chi-squared = 14.97). An increase in the score of this attribute by one with the remaining parameters of the model at a constant level increased the chance of being a ‘heavy user’ by 25.8% (OR = 1.258, 95% CI—Confidence Interval 1.03–1.54).

Consumers for whom ‘ease to prepare’ game was an important attribute of its quality were less likely to be ‘heavy users’, along with an increase in the score by one (OR = 0.723, 95% CI 0.56–0.94).

The greater importance of the ‘national origin’ attribute increased the chance of being a ‘heavy user’ by 60.7% when the attribute’s importance was augmented by one (OR = 1.607, 95% CI 1.22–2.13).

With the increase in the importance of the ‘nutritional value of meat’ attribute, the chance of frequently eating game, i.e., being ‘heavy user’, increased by 31.7% (OR = 1.317, 95% CI 1.12–1.56).

The increase in the importance of ‘low fat content’ as an attribute of quality resulted in an increase in the chance of the frequent consumption of game by 61.1% for each subsequent point in the assessment of this attribute (OR = 1.611, 95% CI 1.23–2.12).

In the group of variables related to emotional motives, statistical significance was confirmed for ‘weight control’ and ‘familiarity’ variables. An increase in the score by one means a 68.8% growth in the possibility being a “heavy user” (OR = 1.688, 95% CI 1.21–2.37) for ‘weight control’ and 25% growth for the “familiarity” variable (OR = 1.250, 95% CI 1.08–2.42) (Table 5).

## 4. Discussion

The results show that the rational motives have a greater impact on game meat choice than emotional reasons. It can be assumed that consumers’ decisions about changing their meat-eating patterns are more rational than emotional, which suggests that the measurable quality characteristics of game meat should be more exposed by producers and sellers on the market. The possibility of increasing the frequency of eating game is greater for the people who pay attention to the rational aspects related to the taste, low fat content, nutritional value and local origin of the meat.

It is known that game stands out among the types of meat available on the market because of its specific taste [57]. Since game meat is mostly derived from animals living freely, its it hard to keep a stable level of meat parameters, as the taste and composition of nutrients in game is determined, among others, by the animal species, its age, sex, health condition, place of living, diet and also by the time and conditions of the acquisition process [58,59]. Despite the fact that the taste of game depends on the type of animal that meat is obtained from [35], the results show that the variable “animal species” is not statistically significant in the prediction model.

In this study, it was shown that consumers who assessed the importance of the taste of game higher than other features were those who consumed it more often, and probably will consume it more often in the future. The results obtained are consistent with recent studies, but the relation between taste significance and eating frequency has not been verified so far [56,58,59,60,61].

The high importance of the aspect of the fat content in the product is not surprising in the context of the recent nutrition trends connected with weight control [62]. In this study, the crucial role of motives related to fat intake, proper nutritional food value and their influence on consumers’ health and condition has been confirmed in both emotional and rational spheres. Buyers paying attention to those aspects fit into the profile of modern consumers who care about the adequate composition of the diet and look for new products to diversify their nutrition [63,64]. The results of the presented study are consistent with the currently observed trend of ‘healthy nutrition’, which is associated with taking action to counteract the occurrence of noncommunicable diseases that are the leading causes of death in the world [65], including obesity, cardiovascular issues, diabetes (type 2) and cancer in some forms [66]. Taking into account the role that the consumption of red, processed meat plays in causing deaths and—on the other hand—the amount of red meat consumed by the average consumer per year, it is reasonable to lower the saturated fatty acid intake by reducing the level of fatty meat consumption [67]. Dutch research shows that consumers are ready to consider changing their meat eating habits by reducing the size of the meat portion or searching for different meat products [68]. The readiness to try organic meat or free range animal meat, as declared by consumers, is a strong signal for game producers to promote their product, showing its nutritional value. The increased health consciousness of consumers has led to a demand for alternative, healthy meat types, with many people opting for low-fat products. Game meat could play a major role as an alternative meat source in the human diet [69].

The study has shown that the probability of eating game several times a year or more often is greater for people declaring the high importance of the ‘national origin of meat’ aspect. The origin of the product is also an important factor of choice for different types of meat [70]. A confirmed origin can also constitute a guarantee of safety for consumers [71,72]. The obtained results suggest that Polish producers of game enjoy consumers’ confidence and local products are perceived as trustworthy, which confirms the opinion of the representatives of the supply sector [73]. Polish hunting traditions, confirmed by the number of people associated with hunting clubs, amounting to over 125 thousand hunters [74], can also increase consumers’ confidence in game. In the context of the results obtained, it must be explained that, according to Polish law, hunters do not participate in the retail trade of game [56]. This legal regulation should be considered in the context of research that proves that buying meat directly from hunters is related to a high level of consumer trust and confidence [49]. It is highly probable that including hunters in the storage chain by allowing them to sell their meat would increase the availability of game meat for the average customer and would cause a greater demand for this kind of meat. Meeting hunters can allow people not only to acquire meat, but also all required information about the product, its features, processing method and usage [75].

An attribute related to quality, indicated as an important factor determining the motives of game consumption, is the ease of its preparation for consumption. It was found that Polish consumers who pay more attention to this attribute tend to eat game less frequently. Similarly, Italian consumers with negative attitudes towards game meat stated difficulties in terms of cooking as a one of the most important factors that stopped them from eating game meat [60]. Tomasevic [35] proved that preparing game can be perceived as an opportunity to present one’s cooking skills. Negative consumer experiences, related with mistakes in the cooking process, can cause a lack of acceptance of the taste of game in general [72]. In Poland, it is widely believed that cooking dishes with game is complicated, time consuming and requires knowledge of game meat specificity [73]. It has also been revealed that the level of game consumption is determined by consumers’ concerns about its safety [76]. Consumers’ concerns are justified, since improperly conducted technological processes may cause not only negative sensory feelings [77], but also adverse effects on consumer health through exposure to toxic substances [78] or pathogens remaining in meat due to the use of an inadequate temperature or time of heat treatment [79,80].

Knowledge about game meat preparation can be transmitted simultaneously with the tradition of game consumption, which is noted by a group of hunters and their families [73]. Eating game is also perceived as a reminder of consumers’ youth and takes them on a journey back to their family home [79]. The importance of the familiarity aspect and its impact on food choice decisions detected in the presented study was also checked and confirmed in the surveys conducted in six European countries, including Poland [81]. The “Consumer First” survey proved that 82% of Polish consumers, while eating meat, seek the traditions and tastes they remember from their childhood [82]. The tastes that were discovered and preserved in youth are those which are most often sought after and demanded in adulthood. Moreover, studies conducted in Belgium, France, Italy, Norway, Poland and Spain confirmed that customers are not open to innovations in relation to traditional food products [83].

## 5. Conclusions

Developing the logistic regression model allowed us to identify the rational and emotional motives that influence consumers’ choices regarding game meat. The results obtained in this research may be interesting not only for academic society, but also for specialists in the game meat sector. Professionals responsible for trade and promotion constantly seek cues about consumers’ needs and expectations. Since this research answers the question about the motives influencing consumers in their behavior towards game meat, it should be perceived as applicable and useful for developing appropriate marketing and information activities within the game market. Producers need to know which aspects of a product are particularly noticeable on labels in relation to advertisement actions.

Regarding the most important motives of game consumption, it seems reasonable to allow consumers to the game dishes and products before purchasing them, since the taste has a major impact on consumers’ attitudes. The product labels should contain information about the origin and nutritional value of the product, which would help consumers decide on their purchase. In terms of usefulness for consumption, it seems necessary to run information programs, which would facilitate the process of overcoming the fear of an unknown product for consumers. Such programs could make use of and present those features that increase the probability of purchasing game, which, as shown by the study results, include the national origin, taste and nutritional value of the game.

In the context of a limited number of studies related to the perception of game, further research should be conducted in the field of demand for this type of meat, with particular attention paid to the perception of game meat quality. Given that food safety is highly important for consumers, it is necessary to carry out wide-ranging information activities that will reduce their concerns about game meat. This may contribute to increasing demand for this meat, as an alternative to the most commonly consumed meat.

## Figures and Tables

**Table 1:** Selected socio-demographic characteristics of the sample.

Specification	Total *N* = 450 (%)	Respondents’ Groups		
		‘Heavy Users’ *N* = 284 (%)	‘Light Users’ *N* = 166 (%)	*p*-Value *
**Gender**				<0.0001
female	186 (41.3)	96 (51.6)	90 (48.4)	
male	264 (58.7)	188 (71.2)	76 (28.8)	
**Education**				0.0094
primary	11 (2.4)	8 (72.7)	3 (27.3)	
vocational/secondary	348 (77.3)	231 (66.4)	117 (33.6)	
higher	91 (20.3)	45 (49.5)	46 (50.5)	
**Age (in years)**				0.1586
25–34	167 (37.1)	112 (67.1)	55 (32.9)	
35–44	122 (27.1)	67 (54.9)	55 (45.1)	
45–54	111 (24.7)	71 (64.0)	40 (36.0)	
55 and over	50 (11.1)	34 (68.0)	16 (32.0)	
**Place of living**				0.0309
town/city	246 (54.7)	144 (58.5)	102 (41.5)	
village	204 (45.3)	140 (68.6)	64 (31.4)	
**Subjective income evaluation**				0.9101
Money is lacking for basic needs.	3 (1.0)	2(66.7)	1 (33.3)	
Enough money for basic needs, but we can’t afford more.	19 (4.2)	14 (73.7)	5 (26.3)	
We can afford everything, but we have to plan larger purchases.	175 (38.9)	110 (62.9)	65 (37.1)	
We can afford everything and we can save.	76 (16.9)	47 (61.8)	29 (38.2)	
Hard to say.	177 (39.3)	111 (62.7)	66 (37.3)	

*—test of independence *x*^2^ at significance level *p* < 0.05.

**Table 2:** Emotional motives for game choice.

It Is Important to Me that Game Meat in My Diet …	Mean	Std. Dev.
**Weight control**	**3.32**	**0.61**
Helps me control my weight	3.17	0.99
Is low in fat	3.71	1.27
Is low in calories	3.23	0.89
**Ethical concern**	**3.54**	**0.65**
Is produced using ethical production methods	3.30	1.17
(e.g., sustainable, animal friendly, without child labor, etc.)		
Is produced in an environmentally friendly way	3.48	1.13
Have a country of origin label	3.44	1.17
**Price**	**3.45**	**0.62**
Is not expensive	3.30	1.12
Is good value for money	3.44	1.12
Is cheap	3.51	1.06
**Natural content**	**3.44**	**0.61**
Contains natural ingredients	3.70	1.19
Contains no artificial ingredients	3.19	1.01
Contains no additives	3.45	0.98
**Convenience**	**3.34**	**0.49**
Is easy buy	3.36	1.15
Is easy to prepare	3.42	0.90
Takes very little time to prepare	3.28	0.94
Can be cooked very easily	3.66	0.89
Is available close to home or the workplace	3.43	0.87
**Health**	**3.38**	**0.47**
Contains a lot of vitamins	3.60	1.20
Contains of iron	3.04	0.91
Keeps me healthy	3.76	1.12
Is nutritious	3.62	1.36
Is high in protein	2.99	1.01
**Sensory appeal**	**3.40**	**0.55**
Smells nice	3.29	1.12
Has a pleasant texture	3.73	1.17
Tastes well	3.64	1.09
Good looking	2.98	1.04
**Familiarity**	**3.24**	**0.51**
Is familiar	3.02	1.01
Is what I usually eat	3.57	0.89
Is like the food I ate when I was a child	3.14	1.02
**Mood**	**3.31**	**0.53**
Makes me feel good	3.64	1.05
Makes me feel special	3.09	0.89
Makes me feel better	3.21	0.87

**Table 3:** Rational motives for game choice connected with quality attributes.

When I Think about the Game Meat Available on the Market, as the Important Feature Determining the Meat Quality I Consider …	Mean	Std. Dev.
Appearance	3.40	0.89
Juiciness	4.14	1.00
Taste	3.50	1.21
Flavor	3.46	1.01
Color	2.95	0.75
Freshness	4.23	0.85
Animal species	3.82	0.77
Slaughter process	3.00	1.00
Low fat	3.61	1.16
Best before date	3.74	0.86
Method of obtaining	3.15	0.91
Ethical production	3.18	0.78
Place of purchase	3.06	0.88
National origin	3.29	0.77
Foreign origin	3.08	0.79
Nutritional value	3.23	0.92
Health value	3.83	1.03
Easy to prepare	3.20	0.79
No shots left in meat	3.83	1.06

**Table 4:** Independent variables included in the statistical analysis.

Emotional Motives		Rational Motives	
It is important to me that game meat in my diet …		When I think about the game meat available on the market, as the important feature determining the meat quality I consider …	
Variables included in the model (statistically significant *)	Variables not included in the model (statistically insignificant)	Variables included in the model (statistically significant *)	Variables not included in the model (statistically insignificant)
Weight Control Familiarity	Ethical concern Price Natural content Convenience Health Sensory appeal Mood	Taste Low fat National origin Nutritional value Easy to prepare	Appearance Juiciness Flavor Color Freshness Animal species Slaughter process Best before date Method of obtaining Ethical production Place of purchase Foreign origin Health value No shots left in meat

*—significance level *p* < 0.05.

**Table 5:** Statistically significant variables and their estimation properties used to develop the logistic regression model.

	Parameter	β	e^β^	95% Wald Confidence Limits		Wald Chi-Squared	*p*-Value
	Intercept	−6.610				21.84	<0.0001
Rational motives	Taste	0.229	1.258	1.028	1.538	14.97	0.0257
	Nutritional value	0.275	1.317	1.115	1.556	10.46	0.0012
	National origin	0.475	1.607	1.216	2.125	11.09	0.0009
	Low fat	0.477	1.611	1.227	2.115	11.76	0.0006
	Easy to prepare	−0.324	0.723	0.558	0.937	6.01	0.0142
Emotional motives	Weight control	0.523	1.688	1.205	2.365	9.25	0.0023
	Familiarity	0.223	1.250	1.082	2.422	5.49	0.0190

β—estimate; e^β^—point estimate (OR-Odds Ratio); *p* < 0.05.

## References

[1] Pearcey S.M., Zhan G.Q. (2018). A comparative study of American and Chinese college students’ motives for food choice. Appetite.

[2] Engel J.F., Blackwell R.D., Miniard P.W. (1995). Consumer Behavior.

[3] Pula K., Parks C.D., Ross C.F. (2014). Regulatory focus and food choice motives. Prevention orientation associated with mood, convenience, and familiarity. Appetite.

[4] Babicz-Zielińska E. (2006). Role of psychological factors in food choice—A review. Pol. J. Food Nutr. Sci..

[5] Acebron L.B., Dopico D.C. (2000). The importance of intrinsic and extrinsic cues to expected and experienced quality: An empirical application for beef. Food Qual. Prefer..

[6] Bilska A., Kowalski R. (2014). Food quality and safety management. LogForum-Sci. J. Logist..

[7] Lawless H. (1995). Dimensions of sensory quality: A critique. Food Qual. Prefer..

[8] McCarthy M., de Boer M., O’Reilly S., Cotter L. (2003). Factors influencing intention to purchase beef in the Irish market. Meat Sci..

[9] Pisula A., Tyburcy A., Dasiewicz K. (2007). Czynniki decydujące o jakości mięsa wołowego. Gospod. Mięsna.

[10] Verbeke W., Van Wezemael L., de Barcellos M.D., Kügler J.O., Hocquette J.-F., Ueland Ø., Grunert K.G. (2010). European beef consumers’ interest in a beef eating-quality guarantee: Insights from a qualitative study in four EU countries. Appetite.

[11] Mascarello G., Pinto A., Parise N., Crovato S., Ravarotto L. (2015). The perception of food quality. Profiling Italian consumers. Appetite.

[12] ISO (2015). Quality Management Systems—Fundamentals and Vocabulary.

[13] Vainio A. (2019). How consumers of meat-based and plant-based diets attend to scientific and commercial information sources: Eating motives, the need for cognition and ability to evaluate information. Appetite.

[14] Bernstein A.M., Sun Q., Hu F.B., Stampfer M.J., Manson J.E., Willett W.C. (2010). Major dietary protein sources and the risk of coronary heart disease in women. Circulation.

[15] Boada L.D., Henríquez-Hernández L.A., Luzardo O.P. (2016). The impact of red and processed meat consumption on cancer and other health outcomes: Epidemiological evidences. Food Chem. Toxicol..

[16] Bradbury K.E., Murphy N., Key T.J. (2020). Diet and colorectal cancer in UK Biobank: A prospective study. Int. J. Epidemiol..

[17] Dehghan M., Mente A., Zhang X., Swaminathan S., Li W., Mohan V., Iqbal R., Kumar R., Wentzel-Viljoen E., Rosengren A. (2017). Associations of fats and carbohydrate intake with cardiovascular disease and mortality in 18 countries from five continents (PURE): A prospective cohort study. Lancet.

[18] Font-i-Furnols M., Guerrero L. (2014). Consumer preference, behavior and perception about meat and meat products: An overview. Meat Sci..

[19] McMichael A.J., Bambrick H.J. (2005). Meat consumption trends and health: Casting a wider risk assessment net. Public Health Nutr..

[20] Vergnaud A.-C., Norat T., Romaguera D., Mouw T., May A.M., Travier N., Luan J., Wareham N., Slimani N., Rinaldi S. (2010). Meat consumption and prospective weight change in participants of the EPIC-PANACEA study. Am. J. Clin. Nutr..

[21] Normy Żywienia 2017 (Nutrition Standards 2017). https://ncez.pl/abc-zywienia-/zasady-zdrowego-zywienia/normy-zywienia-2017.

[22] Limit Red and Processed Meat. Eat No More Than Moderate Amounts of Red Meat and Little, If Any, Processed Meat. https://www.wcrf.org/dietandcancer/recommendations/limit-red-processed-meat.

[23] Willett W., Rockström J., Loken B., Springmann M., Lang T., Vermeulen S., Garnett T., Tilman D., DeClerck F., Wood A. (2019). Food in the Anthropocene: The EAT–Lancet Commission on healthy diets from sustainable food systems. Lancet.

[24] De Boer J., Schösler H., Aiking H. (2017). Towards a reduced meat diet: Mindset and motivation of young vegetarians, low, medium and high meat-eaters. Appetite.

[25] Lea E.J., Crawford D., Worsley A. (2006). Consumers’ readiness to eat a plant-based diet. Eur. J. Clin. Nutr..

[26] Popczyk B., Gwiazdowicz D.J. (2012). Problemy handlu dziczyzną. Probl. Współczesnego Łowiectwa w Polsce. Poznań.

[27] Scholderer J., Kügler J.O., Olsen N.V., Verbeke W. (2013). Meal mapping. Food Qual. Prefer..

[28] Hartmann C., Siegrist M. (2017). Consumer perception and behaviour regarding sustainable protein consumption: A systematic review. Trends Food Sci. Technol..

[29] Fiddes N. (2004). Meat: A Natural Symbol.

[30] Graça J. (2016). Towards an integrated approach to food behaviour: Meat consumption and substitution, from context to consumers. Psychol. Community Health.

[31] Hoffman L.C., Wiklund E. (2006). Game and venison meat for the modern consumer. Meat Sci..

[32] Kwiecińska K., Kosicka-Gębska M., Gębski J., Gutkowska K. (2017). Prediction of the conditions for the consumption of game by Polish consumers. Meat Sci..

[33] Poławska E., Cooper R.G., Jóźwik A., Pomianowski J. (2013). Meat from alternative species–nutritive and dietetic value, and its benefit for human health—A review. CyTA-J. Food.

[34] Strazdina V., Jemeljanovs A., Sterna V., Ikauniece D. (2013). Nutrition value of deer, wild boar and beaver meat hunted in Latvia. 2nd International Conference on Nutrition and Food Sciences IPCBEE.

[35] Tomasevic I., Novakovic S., Solowiej B., Zdolec N., Skunca D., Krocko M., Nedomova S., Kolaj R., Aleksiev G., Djekic I. (2018). Consumers’ perceptions, attitudes and perceived quality of game meat in ten European countries. Meat Sci..

[36] Kudrnáčová E., Bartoň L., Bureš D., Hoffman L.C. (2018). Carcass and meat characteristics from farm-raised and wild fallow deer (Dama dama) and red deer (Cervus elaphus): A review. Meat Sci..

[37] Regulation E.C. (2004). No 853/2004 of the European Parliament and of the Council of 29 April 2004 laying down specific rules for food of animal origin. Off. J. Eur. Union L.

[38] Blaška J., Gašparík J., Šmehýl P., Gondekova M. (2016). Comparison of basic nutritive components of venison in selected species of hoofed game. J. Cent. Eur. Agric..

[39] Okuskhanova E., Assenova B., Rebezov M., Amirkhanov K., Yessimbekov Z., Smolnikova F., Nurgazezova A., Nurymkhan G., Stuart M. (2017). Study of morphology, chemical, and amino acid composition of red deer meat. Vet. World.

[40] Meltzer H.M., Dahl H., Brantsæter A.L., Birgisdottir B.E., Knutsen H.K., Bernhoft A., Oftedal B., Lande U.S., Alexander J., Haugen M. (2013). Consumption of lead-shot cervid meat and blood lead concentrations in a group of adult Norwegians. Environ. Res..

[41] Nationale Verzehrsstudie II. Ergebnisbericht Teil 1. https://www.mri.bund.de/fileadmin/MRI/Institute/EV/NVS_II_Abschlussbericht_Teil_1_mit_Ergaenzungsbericht.pdf.

[42] Ramanzin M., Amici A., Casoli C., Esposito L., Lupi P., Marsico G., Mattiello S., Olivieri O., Ponzetta M.P., Russo C. (2010). Meat from wild ungulates: Ensuring quality and hygiene of an increasing resource. Ital. J. Anim. Sci..

[43] Reinken G. (1998). Production and trade of game and deer meat in Europe. Z. Jagdwiss..

[44] Report of the Scientific Committee of the Spanish Agency for Food Safety and Nutrition (AESAN) in Relation to the Risk Associated with the Presence of Lead in Wild Game Meat in Spain. http://www.aecosan.msssi.gob.es/AECOSAN/docs/documentos/seguridad_alimentaria/evaluacion_riesgos/informes_cc_ingles/LEAD_GAME.pdf.

[45] Siminska E., Bernacka H., Sadowski T. (2011). Sytuacja na światowym i krajowym rynku dziczyzny. Ann. Warsaw Univ. Life Sci. Anim. Sci..

[46] Tolušić Z., Florijančić T., Kralik I., Sesar M., Tolušić M. (2005). Game meat market in Eastern Croatia. Proceedings of the 1st International Symposium “Game and Ecology”.

[47] The National Diet and Nutrition Survey Assesses the Diet, Nutrient Intake and Nutritional Status of the General Population of the UK. https://www.gov.uk/government/collections/national-diet-and-nutrition-survey#archive-of-ndns-reports.

[48] Pickering M., Lawrence J. (2012). Habits and Behaviours of High-Level Consumers of Lead-Shot Wild-Game Meat in Scotland. https://www.foodstandards.gov.scot/publications-and-research/publications/habits-and-behaviours-of-high-level-consumers-of-lead-shot-wild-game-meat-i.

[49] Bodnar K., Szel Hodi M., Skobrak Bodnar E. (2014). Acceptance of the meat of wild ungulates among the hungarian consumers. Agron. Ser. Sci. Res. Stiint. Ser. Agron..

[50] Chardonnet P., Clers B., Fischer J., Gerhold R., Jori F., Lamarque F. (2002). The value of wildlife. Rev. Sci. Tech. Int. Épizooties.

[51] Kwiecińska K., Gębski J., Kosicka-Gębska M. (2018). Dostępność dziczyzny na polskim rynku w kontekście potrzeb konsumentów. Zesz. Nauk. Szk. Głównej Gospod. Wiej. w Warszawie Ekon. i Organ. Gospod. Żywnościowej.

[52] Steptoe A., Pollard T.M., Wardle J. (1995). Development of a measure of the motives underlying the selection of food: The food choice questionnaire. Appetite.

[53] Hosmer D.W., Lemeshow S., Sturdivant R.X. (2013). Applied Logistic Regression.

[54] Hosmer D.W., Lemeshow S. (2000). Applied Logistic Regression.

[55] SAS Institute (1990). SAS/STAT User’s Guide: Version 6.

[56] Kwiecińska K., Kosicka-Gębska M., Gębski J. (2016). Ocena preferencji konsumentów związanych z wyborem dziczyzny. Handel Wewnętrzny.

[57] Hutchison C.L., Mulley R.C., Wiklund E., Flesch J.S. (2010). Consumer evaluation of venison sensory quality: Effects of sex, body condition score and carcase suspension method. Meat Sci..

[58] Hoffman L.C., Kroucamp M., Manley M. (2007). Meat quality characteristics of springbok (Antidorcas marsupialis). 1: Physical meat attributes as influenced by age, gender and production region. Meat Sci..

[59] Neethling J., Hoffman L.C., Muller M. (2016). Factors influencing the flavour of game meat: A review. Meat Sci..

[60] Demartini E., Vecchiato D., Tempesta T., Gaviglio A., Viganò R. (2018). Consumer preferences for red deer meat: A discrete choice analysis considering attitudes towards wild game meat and hunting. Meat Sci..

[61] Sandalj M., Treydte A.C., Ziegler S. (2016). Is wild meat luxury? Quantifying wild meat demand and availability in Hue, Vietnam. Biol. Conserv..

[62] Loebnitz N., Grunert K.G. (2018). Impact of self-health awareness and perceived product benefits on purchase intentions for hedonic and utilitarian foods with nutrition claims. Food Qual. Prefer..

[63] Fowler S.M., Morris S., Hopkins D.L. (2019). Nutritional composition of lamb retail cuts from the carcases of extensively finished lambs. Meat Sci..

[64] Rocha Y.J.P., de Noronha R.L.F., Trindade M.A. (2019). Relations between consumer’s concern with own health and their perception about frankfurters with functional ingredients. Meat Sci..

[65] Decker E.A., Park Y. (2010). Healthier meat products as functional foods. Meat Sci..

[66] Olmedilla-Alonso B., Jiménez-Colmenero F., Sánchez-Muniz F.J. (2013). Development and assessment of healthy properties of meat and meat products designed as functional foods. Meat Sci..

[67] Apostolidis C., McLeay F. (2016). Should we stop meating like this? Reducing meat consumption through substitution. Food Policy.

[68] De Boer J., Schösler H., Aiking H. (2014). “Meatless days” or “less but better”? Exploring strategies to adapt Western meat consumption to health and sustainability challenges. Appetite.

[69] Pophiwa P., Webb E.C., Frylinck L. (2019). A review of factors affecting goat meat quality and mitigating strategies. Small Rumin. Res..

[70] Bernués A., Olaizola A., Corcoran K. (2003). Extrinsic attributes of red meat as indicators of quality in Europe: An application for market segmentation. Food Qual. Prefer..

[71] Becker T. “Country of origin” as a Cue for Quality and Safety of Fresh Meat. Proceedings of the 67th EAAE Seminar.

[72] Radder L., Le Roux R. (2005). Factors affecting food choice in relation to venison: A South African example. Meat Sci..

[73] Kwiecińska K., Kosicka-Gębska M., Gębski J. (2016). Wyzwania dla rozwoju rynku dziczyzny w Polsce. Probl. World Agric. Rol. Światowego.

[74] Central Statistical Office (GUS) (2018). Statistical Yearbook of Forestry.

[75] Kwiecińska K., Kosicka-Gębska M., Gębski J. (2018). Wpływ wybranych źródeł informacji na poziom wiedzy konsumentów o dziczyźnie. Probl. World Agric. Rol. Światowego.

[76] Kwiecińska K., Kosicka-Gębska M., Gębski J. (2015). Poziom bezpieczeństwa jako czynnik warunkujący konsumpcję dziczyzny. Probl. Hig. i Epidemiol..

[77] Radder L. (2002). Restaurants and venison marketing: A South African experience. Food Serv. Technol..

[78] Li J., Dong H., Li X., Han B., Zhu C., Zhang D. (2016). Quantitatively assessing the health risk of exposure to PAHs from intake of smoked meats. Ecotoxicol. Environ. Saf..

[79] Kapperud G., Espeland G., Wahl E., Walde A., Herikstad H., Gustavsen S., Tveit I., Natås O., Bevanger L., Digranes A. (2003). Factors associated with increased and decreased risk of Campylobacter infection: A prospective case-control study in Norway. Am. J. Epidemiol..

[80] Lahou E., Wang X., De Boeck E., Verguldt E., Geeraerd A., Devlieghere F., Uyttendaele M. (2015). Effectiveness of inactivation of foodborne pathogens during simulated home pan frying of steak, hamburger or meat strips. Int. J. Food Microbiol..

[81] Pieniak Z., Verbeke W., Vanhonacker F., Guerrero L., Hersleth M. (2009). Association between traditional food consumption and motives for food choice in six European countries. Appetite.

[82] 82% Polaków w Smaku Mięsa Szuka Tradycji i Smaków Dzieciństwa. https://www.wiadomoscihandlowe.pl/artykuly/82-polakow-w-smaku-miesa-szuka-tradycji-i-smakow-d,58555.

[83] Vanhonacker F., Kühne B., Gellynck X., Guerrero L., Hersleth M., Verbeke W. (2013). Innovations in traditional foods: Impact on perceived traditional character and consumer acceptance. Food Res. Int..

